# FACTORS AFFECTING IMPLEMENTATION OF SOCIALLY ASSISTIVE ROBOTS IN LONG-TERM CARE FACILITIES

**DOI:** 10.1093/geroni/igae098.2559

**Published:** 2024-12-31

**Authors:** Judith Tate, Cathy Maxwell, Miroslava Migovich, Nibraas Khan, Ritam Ghosh, Kelley Colopietro, Abigail Kilpatrick, Nilanjan Sarkar

**Affiliations:** The Ohio State University, Columbus, Ohio, United States; University of Utah College of Nursing, Salt Lake City, Utah, United States; Vanderbilt University, Nashville, Tennessee, United States; Vanderbilt University, Nashville, Tennessee, United States; Vanderbilt University, Nashville, Tennessee, United States; Vanderbilt University, Nashville, Tennessee, United States; The Ohio State University, Columbus, Ohio, United States; Vanderbilt University, Nashville, Tennessee, United States

## Abstract

Older adult residents in long term care (LTC) often experience apathy. Technology can provide support for addressing the older adults’ psychosocial needs. However, introducing new technology into LTCs can be challenging. As part of a randomized clinical trial on socially assistive robots (SAR) in LTCs to engage older adult dyads in activities, we examined determinants to implementation. Seventeen LTCs participated in the study, ranging in size from 24 to 132 participants; 53% were not-for-profit. All robot sessions were conducted by trained research staff. Each participant attended 16 sessions averaging 30 minutes over 8 weeks; half with an animal SAR and half with a humanoid SAR. Research assistants noted observations of the SAR, participant, staff, and facility environment and conducted interviews at the conclusion of the experiment. Sixty older adults participated (62% women, ages 60 to 98). Common SAR barriers included internet connections, equipment breakage, and speaker volume. Participant barriers included paranoia, dislike of partner, hearing and vision, level of cognitive impairment, and acute illness. Staff issues consisted of workflow issues and knowledge of the research. Facility issues included competing demands that interrupted scheduling and rollout, physical area for conduct of experiments, noise and lighting, and communication among key staff. Successful implementation and sustainability of new technology, including SAR, will depend upon addressing multiple barriers at the resident, staff, and facility levels.

